# Emergence of extensive drug resistance and high prevalence of multidrug resistance among clinical *Proteus mirabilis* isolates in Egypt

**DOI:** 10.1186/s12941-024-00705-3

**Published:** 2024-05-24

**Authors:** Maggi ElTaweel, Heba Shehta Said, Rasha Barwa

**Affiliations:** Department of Microbiology and Immunology, Faculty of Pharmacy, Mansoura University, Mansoura, 35516 Egypt

**Keywords:** *Proteus mirabilis*, MDR, XDR, ESBLs, AmpCs, Carbapenemases, PMQR, Integrons, ERIC typing

## Abstract

**Background:**

*Proteus mirabilis* is an opportunistic pathogen that has been held responsible for numerous nosocomial and community-acquired infections which are difficult to be controlled because of its diverse antimicrobial resistance mechanisms.

**Methods:**

Antimicrobial susceptibility patterns of *P. mirabilis* isolates collected from different clinical sources in Mansoura University Hospitals, Egypt was determined. Moreover, the underlying resistance mechanisms and genetic relatedness between isolates were investigated.

**Results:**

Antimicrobial susceptibility testing indicated elevated levels of resistance to different classes of antimicrobials among the tested *P. mirabilis* clinical isolates (*n* = 66). ERIC-PCR showed great diversity among the tested isolates. Six isolates (9.1%) were XDR while all the remaining isolates were MDR. ESBLs and AmpCs were detected in 57.6% and 21.2% of the isolates, respectively, where *bla*_TEM_, *bla*_SHV_, *bla*_CTX−M_, *bla*_CIT−M_ and *bla*_AmpC_ were detected. Carbapenemases and MBLs were detected in 10.6 and 9.1% of the isolates, respectively, where *bla*_OXA−48_ and *bla*_NDM−1_ genes were detected. Quinolone resistant isolates (75.8%) harbored *acc(6')-Ib-cr*, *qnrD, qnrA*, and *qnrS* genes. Resistance to aminoglycosides, trimethoprim-sulfamethoxazole and chloramphenicol exceeded 80%. Fosfomycin was the most active drug against the tested isolates as only 22.7% were resistant. Class I or II integrons were detected in 86.4% of the isolates. Among class I integron positive isolates, four different gene cassette arrays (*dfrA17- aadA5*, *aadB-aadA2*, *aadA2-lnuF*, and *dfrA14-arr-3-bla*_OXA−10_-*aadA15*) and two gene cassettes (*dfrA7* and *aadA1*) were detected. While class II integron positive isolates carried four different gene cassette arrays (*dfrA1-sat1-aadA1*, *estXVr-sat2-aadA1*, *lnuF- dfrA1-aadA1*, and *dfrA1-sat2*).

**Conclusion:**

*P. Mirabilis* ability to acquire resistance determinants via integrons may be held responsible for the elevated rates of antimicrobial resistance and emergence of XDR or even PDR strains limiting the available therapeutic options for management of infections caused by those strains.

**Supplementary Information:**

The online version contains supplementary material available at 10.1186/s12941-024-00705-3.

## Background

*Proteus mirabilis* is Gram-Negative, facultative anaerobe that belongs to family Morganellaceae. It is ubiquitous in nature and a member of the gastrointestinal flora of animals and human. However, it is held responsible for many nosocomial and community acquired outbreaks all over the world including urinary and respiratory tract infections, foot ulcers of the diabetic patients, and wide range of other infections [1].

Misuse or non-specific use of antibiotics has led to increased levels of drug resistance and wide spread of various resistance genes among clinical *P. mirabilis* isolates. Besides, *P. mirabilis* is characterized by intrinsic resistance to tetracycline, tigecycline, and polymyxins [2]. β-lactam antibiotics, including penicillins and cephalosporins, and carbapenems is considered the first choice for treatment of infections caused by *P. mirabilis.* One of the most common resistance mechanisms is the enzymatic hydrolysis of β-lactam antibiotics [3]. Structural and functional classification of β-lactamases have a critical role in the adequate choice of appropriate antimicrobial agent [4]. Prevalence of carbapenem resistance is relatively low, although it is increasing with time [5].

Recently, elevated levels of resistance to quinolones and aminoglycosides were reported worldwide [6, 7]. In addition, folate pathway inhibitors, nitrofurans, and even fosfomycin resistance are increasingly reported [8–10].

Most of resistance determinants are carried on integron’s that can be transferred by plasmids, transposons and other mobile genetic elements. Therefore, it is considered a major cause for the transfer of drug resistance traits among different bacterial pathogens, especially in family *Enterobacteriaceae.* More than 130 integron’s gene cassette arrays of various resistance genes to different classes of antibiotics have been identified [11].

Clinicians may face very limited therapeutic options, due to spread of multidrug-resistant (MDR), emergence of extensive drug resistant (XDR) and even pandrug resistant (PDR) strains [12]. Therefore, the aim of the present study is to assess the prevalence of resistance to different classes of antimicrobial agents among *P. mirabilis* isolates collected from different clinical sources from Mansoura University Hospitals, Egypt. Moreover, molecular detection of underlying resistance mechanisms and genetic relatedness among collected isolates was unveiled.

## Materials and methods

### Bacterial isolates

Bacterial isolates were collected from Mansoura University Hospitals from different clinical sources between September 2021 and January 2022. Isolates were identified as *P. mirabilis* according to standard microbiology and molecular methods [13].

### Antimicrobial susceptibility pattern of *P. mirabilis* clinical isolates

Kirby Bauer disk diffusion method was used to assess *P. mirabilis* antimicrobial susceptibility profile using Mueller Hinton agar plates [14]. Interpretation of the results was performed according to the recommendations of Clinical and Laboratory Standard Institute [15]. Antimicrobial discs of various antimicrobial categories (Bioanalyze ® products, Turkey) have been used to assess the resistance profile of the tested *P. mirabilis* clinical isolates (Table S1).

Bacterial isolates were classified into MDR, XDR and PDR. MDR is recognized as being non susceptible to at least one antimicrobial agent in three antimicrobial classes or more. While XDR is being resistant to at least one antimicrobial agent in all antimicrobial classes but susceptible to two or fewer. PDR is recognized as being resistant to all agents in all different antimicrobial classes [16].

### Phenotypic detection of β-lactamases

#### Acidimetric method for β-lactamases detection

β-lactamases hydrolyze β-lactam ring resulting in generating a carboxyl group which acidifies un-buffered systems. The resulting acidity was tested in 96-wells microtiter plates using benzylpenicillin as substrate and phenol red as a pH indicator [17].

#### Detection of extended spectrum β-lactamases (ESBLs)

Combination disk method (CDM) was used as previously described [15]. The zone of inhibition of cefotaxime (30 µg) and ceftazidime (30 µg) discs alone was measured and compared with that of cefotaxime/clavulanate (30 µg / 10 µg), and ceftazidime/ clavulanate (30 µg / 10 µg) discs, respectively. An increase in the inhibition zone (≥ 5 mm) of either or both anti-microbial agents when combined with clavulanate indicates ESBLs production.

#### Detection of plasmid mediated cephalosporinases (AmpCs)

Streaking method on MacConkey agar plates was employed as described previously [18]. In brief, sensitive *E. coli DH5α* strain lawn was inoculated on the surface of MacConkey agar plates. Cefoxitin disc (30 µg) was applied in the center of the plate and the tested isolates were streaked as a line, away from the cefoxitin disc by 5 mm. Isolates that distorted cefoxitin inhibition zone (clover leaf-like shape) was considered AmpC producers.

#### Detection of carbapenemases

Modified Hodge test (MHT) was used according to CLSI recommendations [15]. Sensitive *E. coli DH5α* strain lawn culture was inoculated on MacConkey agar plates [19]. Meropenem disc (10 µg) was applied in the center of the plate. The tested isolates were streaked as a line, 5 mm away from the disc. Distortion of the meropenem inhibition zone (clover leaf-like shape) indicates carbapenemase production [20].

#### Detection of metallo β‑lactamases (MBLs)

Combined disk synergy test (CDST) was used to as described previously. Briefly, two disks of IPM (10 µg) were placed on inoculated Mueller Hinton agar plate and then 2 µL of 0.5 M EDTA was added to one of them [21]. If inhibition zone of IPM/EDTA increased by ≥ 5 mm compared with that of IPM alone, the isolate was considered MBLs producer [15].

### Molecular detection of resistance determinants

#### Extraction of DNA

DNA was extracted by boiling method as previously described [22]. Amplification of *UreC* gene was used for confirming identification of *P. mirabilis* isolates (Table 1) [23].

**Table 1:** Oligonucleotides used in this study

Target Gene		Nucleotide Sequence (5’- 3’)		Amplicon size (bp)	Annealing Temp (°C)	Reference
1. β-lactam resistance encoding genes						
1.1. ESBLs encoding genes						
**Class A β-lactamases**						
*bla*_SHV_	Fw	ACTATCGCCAGCAGGATC		356	53.5	59
	Rv	ATCGTCCACCATCCACTG				
*bla*_TEM_	Fw	GATCTCAACAGCGGTAAG		786	54	59
	Rv	CAGTGAGGCACCTATCTC				
*bla*_CTX−M_	Fw	ATGTGCAGYACCAGTAARGT		593	50	60
	Rv	TGGGTRAARTARGTSACCAGA				
*bla*_GES_ *(GES-1 to GES-9 and GES-11)*	Fw	AGTCGGCTAGACCGGAAAG		399	60	61
	Rv	TTTGTCCGTGCTCAGGAT				
*bla*_PER_ *(PER-1 & PER-3)*	Fw	GCTCCGATAATGAAAGCGT		520	60	61
	Rv	TTCGGCTTGACTCGGCTGA				
*bla*_VE*B*_ *(VEB-1 to VEB-6)*	Fw	CATTTCCCGATGCAAAGCGT		648	60	61
	Rv	CGAAGTTTCTTTGGACTCTG				
**Class D β-lactamases**						
*bla*_OXA-1-like_ *(OXA-1, OXA-4 & OXA-30)*	Fw	GGCACCAGATTCAACTTTCAAG		564	58	61
	Rv	GACCCCAAGTTTCCTGTAAGTG				
**1.2. AmpC encoding genes (Class C A β-lactamases)**						
*bla*_AmpC_	Fw	ACACGAGTTTGCATCGCCTG		254	68	62
	Rv	CTGAACTTACCGCTAAACAGTGGAAT				
*bla*_CIT−M_	Fw	TGGCCAGAACTGACAGGCAAA		462	55	62
	Rv	TTTCTCCTGAACGTGGCTGGC				
*bla*_Fox−1_	Fw	GCAAACCAGCAATACCATCCA		642	60	62
	Rv	GCTCACCTTGTCATCCAGCTC				
*bla*_ACC−1_	Fw	AGCTGTTATCCGTGATTACCTGTCT		248	60	62
	Rv	AGCGAACCCACTTCAAATAACG				
*bla*_ACT−1_	Fw	CATGCTGGATCTGGCAACCT		343	60	62
	Rv	CTTCAGCGTCCAGCATTCCT				
*bla*_FOX_ *(FOX-1 to FOX-5)*	Fw	CTACAGTGCGGGTGGTTT		162	55	61
	Rv	CTATTTGCGGCCAGGTGA				
*bla*_MOX_ *(MOX1, MOX2*, *CMY-CMY-8 to CMY 11 and CMY19)*	Fw	GCAACAACGACAATCCATCCT		895	60	61
	Rv	GGGATAGGCGTAACTCTCCCAA				
**1.3. Carbapenemases encoding genes**						
**Class A (Serine enzymes)**						
*bla*_KPC_ (KPC-1 to KPC-5)	Fw	CATTCAAGGGCTTTCTTGCTGC		538	55	61
	Rv	ACGACGGCATAGTCATTTGC				
**Class B (MBLs)**						
*bla*_IMP_	Fw	CATGGTTTGGTGGTTCTTGT		448	57	55
	Rv	ATAATTTGGCGGACTTTGGC				
*bla*_VIM−1_	Fw	TGTTATGGAGCAGCAACGATG		920	56	63
	Rv	AAAGTCCCGCTCCAACGATT				
*bla*_NDM−1_	Fw	GGTTTGGCGATCTGGTTTTC		621	52	64
	Rv	CGGAATGGCTCATCACGATC				
**Class D (Oxacillinases)**						
*bla*_OXA−48−like_	Fw	GCTTGATCGCCCTCGATT		281	57	61
	Rv	GATTTGCTCCGTGGCCGAAA				
**2. Quinolones resistance encoding genes**						
*qnrA* *(qnrA1 to qnrA6)*	Fw	AGAGGATTTCTCACGCCAGG		580	60	65
	Rv	TGCCAGGCACAGATCTTGAC				
*qnrB* *(qnrB1 to qnrB6)*	Fw	GGMATHGAAATTCGCCACTG		264	60	66
	Rv	TTTGCYGYYCGCCAGTCGAA				
*qnrS* *(qnrS1 to qnrS2)*	Fw	GCAAGTTCATTGAACAGGCT		428	60	65
	Rv	TCTAAACCGTCGAGTTCGGCG				
*qnr C*	Fw	GGGTTGTACATTTATTGAATC		447	55	67
	Rv	TCCACTTTACGAGGTTCT				
*qnr D*	Fw	CGAGATCAATTTACGGGGAATA		582	55	67
	Rv	AACAAGCTGAAGCGCCTG				
*qepA*	Fw	CGTGTTGCTGGAGTTCTTC		403	52	68
	Rv	CTGCAGGTACTGCGTCATG				
*oqxA*	Fw	GACAGCGTCGCACAGAATG		339	57	69
	Rv	GGAGACGAGGTTGGTATGGA				
*oqxB*	Fw	CGAAGAAAGACCTCCCTACCC		240	57	69
	Rv	CGCCGCCAATGAGATACA				
*aac(6'*)*-Ib-cr*	Fw	TTGCGATGCTCTATGAGTGGCTA		482	60	70
	Rv	CTCGAATGCCTGGCGTGTTT				
**3. Integrons and Variable regions**						
**Integrons constant regions**						
*IntI1*	Fw	GGTCAAGGATCTGGATTTCG		483	58	71
	Rv	ACATGCGTGTAAATCATCGTC				
*IntI2*	Fw	CACGGATATGCGACAAAAAGGT		788	58	71
	Rv	GTAGCAAACGAGTGACGAAATG				
**Variable region of integron I**						
*5’CS*	Fw	GGCATCCAAGCAGCAAG		Variable	58	71
*3’CS*	Rv	AAGCAGACTTGACCTGA				
**Variable region of integron II**						
*attI2*	Fw	GACGGCATGCACGATTTGTA		Variable	58	71
*orfX*	Rv	GATGCCATCGCAAGTACGAG				
**4. Molecular Identification**						
*UreC*	Fw	GTTATTCGTGATGGTATGGG		317	56.2	58
	Rv	ATAAAGGTGGTTACGCCAGA				
**5. Molecular Typing**						
ERIC-2 typing	2	AAGTAAGTGACTGGGGTGAGCG	Variable		48	73

bp: base pair, Fw: forward primer, Rv: reverse primer

#### Detection of antimicrobial resistance encoding genes

Prevalence of antimicrobial resistance determinants including β‑lactams [24–30] and quinolones [31–36] resistance encoding genes among the tested *P. mirabilis* clinical isolates was detected via PCR using DreamTaq™ Green PCR master mix (Thermo Scientific, USA). PCR was performed in ProFlex™ PCR System (Cat. No. 4,484,073, Applied Biosystems™, USA) with programmed cycling conditions: initial denaturation (95 °C / 5 min), followed by 35 cycles of denaturation (95 °C / 30s), annealing (specified temperature (Table 1) / 30s) and extension step (72 °C / 60 s), then final extension (72 °C / 5 min). PCR products were analyzed using agarose gel electrophoresis (1.2%), stained with ethidium bromide, and their sizes were confirmed under ultraviolet light by comparison with appropriate DNA markers (Gene Ruler 100 bp or Gene Ruler 100 bp plus, Thermo Fisher Scientific Tm, UK).

#### Integrase genes and gene cassettes amplification

Amplification of integrase gene was performed using primers for *intI1 and intI2* to detect class I and class II integrons, respectively [37]. Variable regions (gene cassettes) in isolates harboring class I integrons were amplified using *5’CS* and *3’CS* primers and class II integrons were amplified using *attI2* and *orfX* primers (Table 1) [37]. PCR conditions were as follows; an initial denaturation (95 °C / 5 min), followed by 35 cycles of denaturation (95 °C / 1 min), annealing (58 °C / 1 min), and extension (72 °C / 2 min). Last step was final extension (72 °C / 10 min).

#### Sequencing and characterization of variable region’s gene cassettes

Amplicons of the same size of the variable region of class I and class II integrons (≥ 800 bp) were compared by restriction analysis with *HinfI* (TaKaRa, Japan). At least one representative of each type was sequenced using an automated sequencer (ABI Prism 3100), as described previously [38]. BLAST against the GenBank database (http://blast.ncbi.nlm.nih.gov/Blast.cgi) was performed for sequence comparison, and annotation. Nucleotide sequences of integrons’ variable regions were deposited in GenBank.

### Molecular typing

Enterobacterial repetitive intergenic consensus PCR (ERIC-PCR) was used for detecting the genetic relatedness of the tested *P. mirabilis* clinical isolates using specific primers (Table 1) [39]. PCR was conducted starting with initial denaturation (95 °C/5 min), then 35 cycles of denaturation (95 °C / 1 min), annealing (48 °C / 1 min), and extension step (72 °C / 5 min). Finally, termination of the reaction by a final extension step (72 °C / 5 min) [40]. The amplified products were separated on 2% agarose gel. The resulting banding patterns of ERIC-PCR were analyzed by Gel J software version 2.0 [41]. Unweighted pair-group method with arithmetic mean (UPGMA) and Jaccard’s coefficient were used for similarity clustering analysis. Clinical *P. mirabilis* isolates with a similarity coefficient ≥ 85% were considered genetically related [42].

### Statistical analysis

R Studio (version 1.3.1093) was used for data analysis. Comparisons of frequencies and association phenotypic of and genotypic features were analyzed by contingency tables using the chi-square test (*P* < 0.05). Heat map was used for data graphing and visualization.

## Results

### Collection and identification of *P. mirabilis* clinical isolates

Sixty-six clinical isolates were collected and identified as *P. mirabilis* according to standard microbiological and molecular procedures (Table S1). Isolates were collected from different clinical sources including urine (24 isolates, 36.4%), diabetic foot lesions (12 isolates, 18.2%), sputum (7 isolates, 10.6%), T-Tube drain (6 isolates, 9.1%), bedsores and wound swabs (6 isolates, 9.1%), burn swab (5 isolates, 7.6%), blood (3 isolates, 4.5%), thigh boils swabs (2 isolates, 3%), and aspirate swab (1 isolate, 1.5%).

Clinical isolates of *P. mirabilis* appeared as scattered Gram-negative rods with characteristic fishy odor and strong swarming motility on nutrient agar. Identification of *P. mirabilis* isolates was performed according to their biochemical profile including positive phenyl alanine deaminase test, negative indole test, positive citrate utilization test, positive Voges-Proskauer (VP) and methyl red (MR) tests, and non-lactose fermentation on MacConkey’s agar media. On triple sugar iron agar slants, isolates showed alkaline red slant with acidic butt (indicative of glucose fermentation only) and heavy black precipitate (indicative of hydrogen sulfide production). Moreover, the gene coding for urease enzyme (*UreC*) was successfully amplified in all *P. mirabilis* clinical isolates confirming its identification.

### Antibiotic sensitivity pattern of *P. mirabilis* clinical isolates

**Fig. 1:**
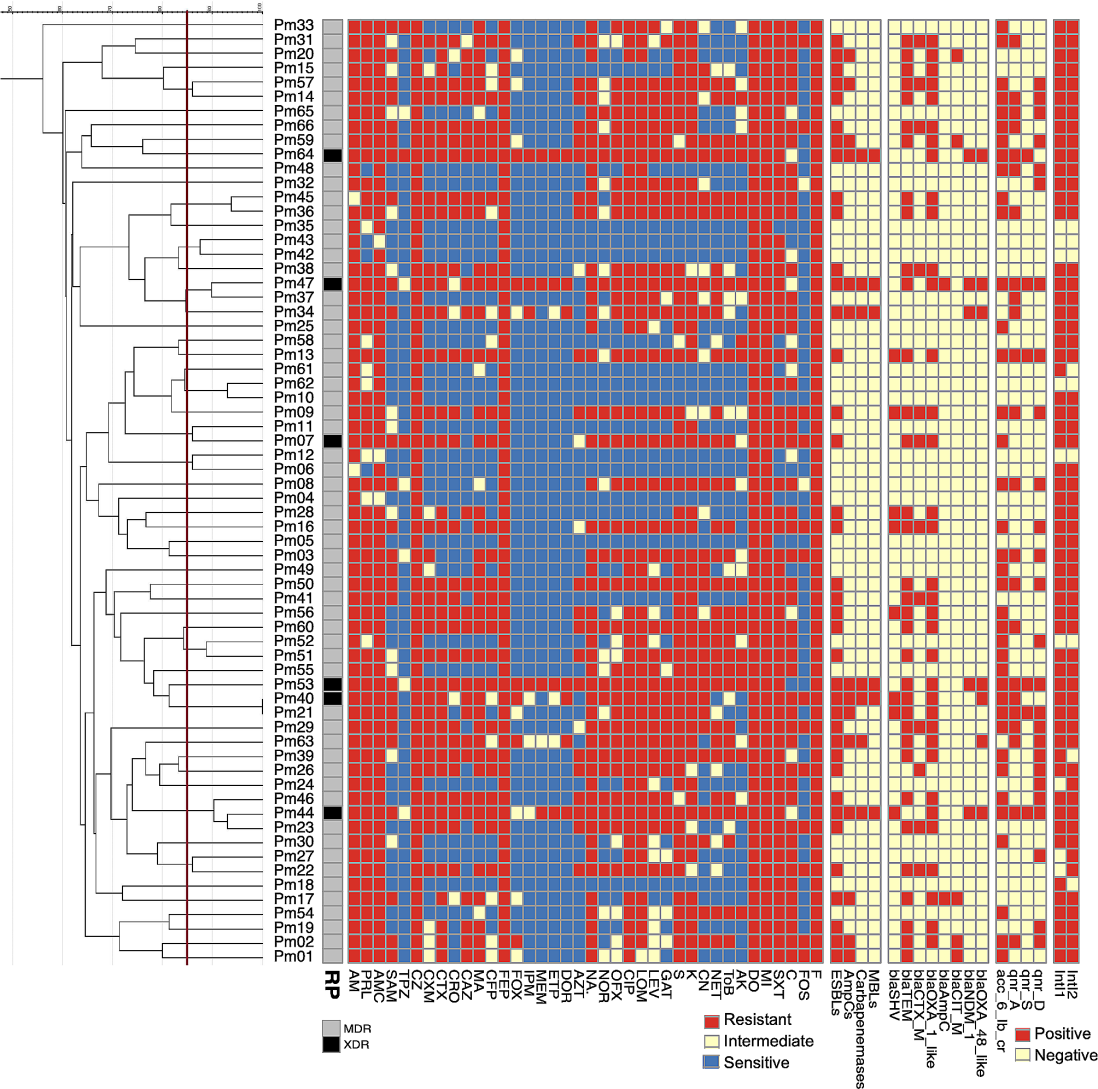
Dendrogram constructed using ERIC-PCR patterns of *P. mirabilis* clinical isolates. Banding patterns were analyzed by using Gel J software version 2.0. Analysis of similarity clustering was performed by using UPGMA and Jaccard’s coefficient. The vertical line is a hypothetical line illustrating 85% similarity. Heatmap representing resistance profile of each isolate to different classes of antibiotics (red = resistant, yellow = intermediate, and blue = sensitive), and resistance determinants (red = positive, and yellow = negative) was added for comparison between isolates

Antibiotic sensitivity testing of *P. mirabilis* clinical isolates indicated elevated rates of resistance to ampicillin, amoxicillin-clavulanic acid, cefazolin, cefuroxime, cefepime, trimethoprim-sulfamethoxazole, nitrofurantoin and Chloramphenicol (Fig. 1 and Table S2). Resistance to third generation cephalosporins ranged from 44 to 68%, while resistance to carbapenems was less than 11%. High rates of resistance to fluoroquinolones (53 to 76%) and aminoglycosides (45 to 82%) were observed, while fosfomycin resistance was detected in 23% of the tested isolates. Based on the resistance profile of *P. mirabilis* clinical isolates against the tested antimicrobials, 60 isolates (90.9%) were MDR, 6 isolates (9.1%) were XDR, and none of the isolates were PDR (Fig. 1 and Table S1).

### Phenotypic detection of β-lactamases

#### Detection of β-lactamases

Acidimetric test was used for general detection of β-lactamases. The results showed that 38 isolates (57.6%), including all the XDR isolates, were β-lactamase producers (Table S1).

#### Detection of ESBLs

Based on CDM (Fig. S1), 38 isolates (57.6%) were ESBLs producers (Fig. 1 and Table S1).

#### Detection of AmpC

Fourteen isolates (21.2%) showed distortion of cefoxitin inhibition zone (clover leaf-like shape, Fig. S1) and were considered positive AmpC producers (Fig. 1 and Table S1).

#### Detection of MBLs

Six isolates (9.1%) showed increase in inhibition zone of Imipenem/EDTA by ≥ 5 mm compared with that of IPM alone in CDST (Fig. S1) and were considered MBLs producers (Fig. 1 and Table S1).

#### Detection of carbapenemases

Seven isolates (10.6%) distorted the inhibition zone around meropenem disc (clover leaf like shape, Fig. S1) in MHT indicating carbapenemases production (Fig. 1 and Table S1).

### Molecular detection of resistance determinants

#### Detection of β-lactamases encoding genes

Among the tested *P. mirabilis* clinical isolates, *bla*_OXA−1−like_ (class D β-lactamases) was the most predominant as it was detected in 35 isolates (53%) (Fig. 1, Fig. S2 and Table S1). Regarding Class A β-lactamases, *bla*_TEM_ was detected in 34 isolates (51.5%), *bla*_SHV_ in 8 isolates (12.1%), *bla*_CTX−M_ in 12 isolates (18.2%). While *bla*_*GES*_, *bla*_*PER*_, and *bla*_*VEB*_ genes were not detected among the tested isolates.

#### Detection of AmpC encoding genes

Among the tested genes, *bla*_*CIT−M*_ and *bla*_*AmpC*_ genes were detected in 5 isolates (7.6%) and 2 isolates (3%), respectively (Fig. 1, Fig. S2 and Table S1). While *bla*_ACC−1_, *bla*_ACT−1_, *bla*_Fox_, and *bla*_MOX_ genes were not detected among the tested isolates.

#### Detection of carbapenemases and MBLs encoding genes

*bla*_OXA−48−like_ gene (class D β-lactamases) was detected in all carbapenem resistant *P. mirabilis* isolates (7 isolates, 10.6%), while *bla*_KPC_ gene (class A β-lactamases) was not detected (Fig. 1, Fig. S2 and Table S1). Among MBLs encoding genes, *bla*_NDM−1_ was detected in 5 MBLs producing isolates (7.6%). While *bla*_VIM−1_ and *bla*_IMP_ genes were not detected.

#### Detection of quinolone resistance encoding genes

Analysis of plasmid mediated quinolone resistance (PMQR) genes showed that *acc (6’)-Ib-cr* gene was detected in 38 isolates (57.6%). Regarding *qnr* encoding genes, *qnrD* was detected in 26 isolates (39.4%), *qnrA* in 23 isolates (34.8%), and *qnrS* in 6 isolates (9.1%) (Fig. 1, Fig. S2 and Table S1). Other qnr encoding genes (*qnrB* and *qnrC)* and *qepA, oqxA*, and *oqxB* were not detected.

### Detection of integrons and their gene cassettes

**Fig. 2:**
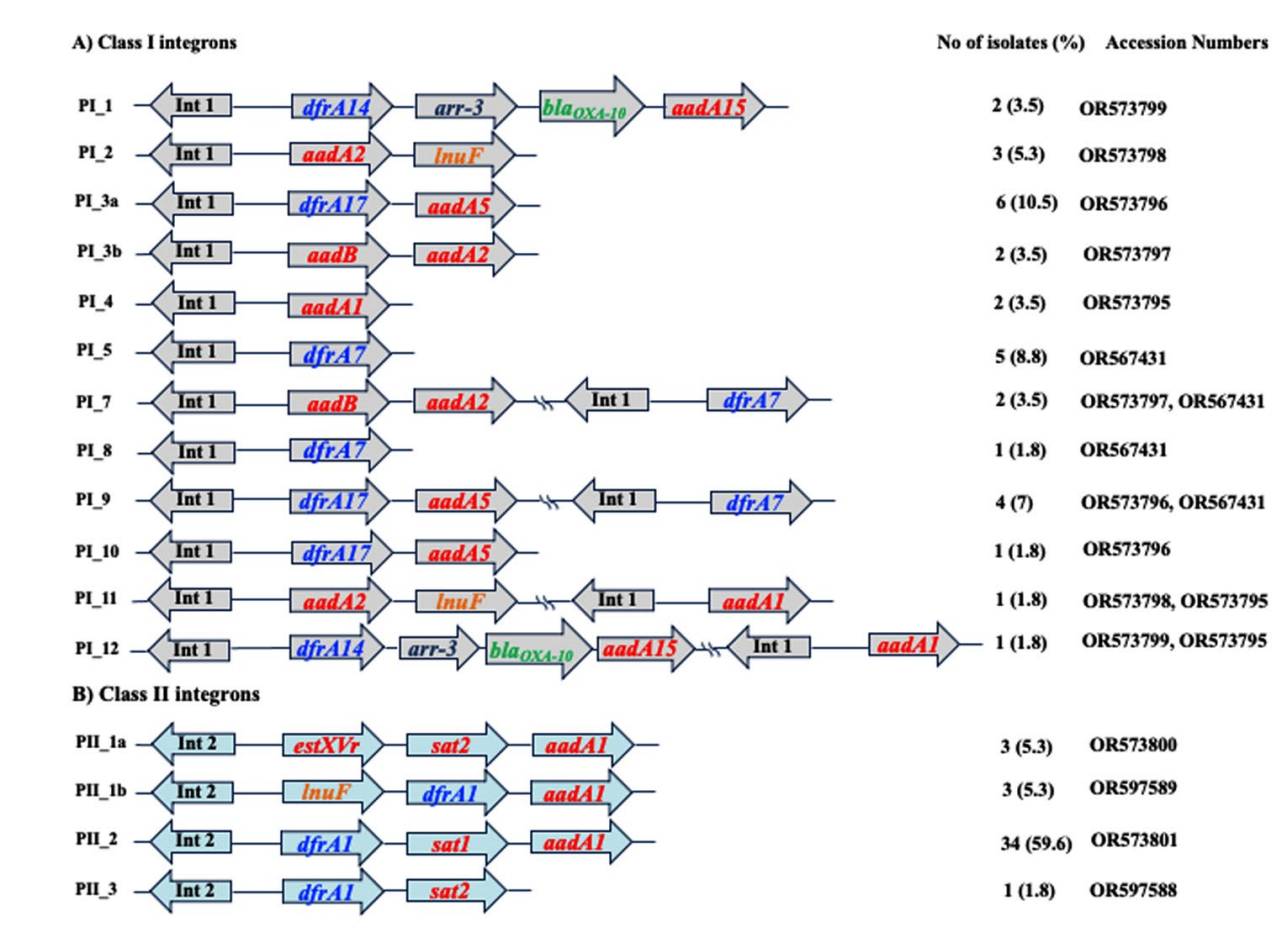
Schematic representation of class I and II integrons variable region’s (gene cassettes/arrays) detected among the tested *P. mirabilis* clinical isolates

Integrons Class I and II were screened among all the tested *P. mirabilis* clinical isolates. Fifty-seven isolates (86.4%) harbored either class I or class II integrons. Fifty-one isolates (77.3%) harbored both class I and II integrons, while six isolates (9.1%) lacked both (Fig. S3 and Table S1). The variable regions of class I and class II integrons were successfully amplified in 42 isolates (73.7%) and 54 isolates (94.7%), respectively. The size of the variable region ranged from 200 to 3000 bp (Fig. S3 and Table S1). Nucleotide sequences of class I integron’s variable region were deposited in GenBank (accessions no. OR567431, and OR573795:OR573799). Four different gene cassette arrays, *dfrA17-aadA5* (11 isolates), *aadB-aadA2* (4 isolates), *aadA2-lnuF* (4 isolates), and *dfrA14-arr-3-bla*_OXA−10_*-aadA15* (3 isolates), and two different gene cassettes, *dfrA7* (12 isolates), and *aadA1* (4 isolates), were detected. While four different gene cassette arrays, *dfrA1-sat1-aadA1* (34 isolates), *estXVr-sat2-aadA1* (3 isolates), *lnuF-dfrA1-aadA1* (3 isolates), and *dfrA1-sat2* (1 isolate), were detected in class II integron’s variable regions (Fig. 2 and Table S1). Nucleotide sequences were deposited in GenBank (accessions no. OR597588, OR597589, OR573800, and OR573801).

Correlation between resistance to aminoglycosides tested and folate pathway inhibitors (trimethoprim/sulfamethoxazole) and their resistance determinants (*aadA*, *sat* and *dfrA* variants) carried by either class I or class II integrons (chi-square test, *P* < 0.5). Most isolates lacking integrons (class I and class II) were sensitive to the tested aminoglycosides and folate pathway inhibitors.

### Molecular typing

ERIC-PCR typing method demonstrated enormous diversity among the tested *P. mirabilis* clinical isolates. Some isolates were considered genetically related as they showed a similarity coefficient higher than 85% (Fig. 1). Moreover, it showed great diversity among isolates that showed XDR profile and were considered genetically unrelated.

## Discussion

Management of infectious diseases is of great importance for human health, especially with the continuous increase of MDR and the emergence of XDR or even PDR [5, 22, 43–45].

Therefore, evaluation of the local antimicrobial resistance patterns and underlying resistance determinants is fundamental for the implementation of effective stewardship programs in each country/region.

Among *Enterobacterales*, *K. pneumoniae, E. coli* and *P. mirabilis* were held responsible for most of hospital and community-acquired infections. *P. mirabilis* caused several nosocomial and community-acquired outbreaks in different regions of the world [46]. It does not produce any chromosomally encoded β-lactamases resulting in full susceptibility to all β-lactams for a wild-type phenotype and it is generally susceptible to fluoroquinolones [4]. However, strains resistant to different antibiotics are increasingly reported, which complicates the treatment of infections caused by Proteus spp [12].

In this study, 57.6% of the tested isolates were ESBLs producers which coincide with reports in different regions of the world [47–49]. In Egypt, low rate ESBLs production (28.3%) has been reported but a recent study recorded a higher rate (51.7%) [9, 10]. *bla*_TEM_, *bla*_CTX−M_, and *bla*_SHV_ genes were detected in the tested isolates. Recent studies indicated similar findings in Egypt [9, 10, 12] and worldwide [3, 50–52].

In Croatia, *bla*_TEM_ and *bla*_PER_ genes were detected [53, 54], while *bla*_CTX−M2_ was the most prevalent ESBLs encoding gene in Japan [55].

AmpC cephalosporinases were held responsible for nosocomial outbreaks and failure of treatment.

AmpC production was detected in 21.2% of the tested isolates which coincide with previous studies [49, 56]. In Egypt, low AmpC production was previously recorded (3.8%), but a recent study reported higher AmpC production (34.5%) [10, 57]. *bla*_*CIT−M*_ and *bla*_*AmpC*_ genes were detected among the tested isolates as previously reported in Egypt [10, 57]. In Singapore and Bahrain, only *bla*_*CIT−M*_ was detected [58, 59], while a recent study in Nigeria showed that 66.7% of the isolates carried *bla*_*AmpC*_ gene [60].

Carbapenems remain one of the last resort antibiotics for treatment of severe infection especially those caused by ESBLs producing Enterobacterales. Globally resistance of carbapenem in *P. mirabilis* is relatively low, although it tends to increase with time [10, 61]. Among the tested isolates, 10.6% were carbapenem resistant and *bla*_OXA−48_ and *bla*_NDM−1_ genes were detected. A recent study in Egypt recorded *bla*_KPC_, and *bla*_NDM−1_ [12], while another study recorded *bla*_oxa_ and *bla*_VIM_ genes [10]. In general, *bla*_KPC_, *bla*_NDM−1_, *bla*_VIM_ and *bla*_OXA−48_ are the most predominant in Europe, *bla*_KPC_ in United States and *bla*_IMP_ in Japan [62, 63].

Quinolones is a promising, relatively safe substitute to β-lactams in case of their resistance. However, high quinolone resistance (75.8%) was detected among the tested isolates. The prevalence of resistance determinants was studied, where acetylation by *acc (6’)-Ib-cr* gene was the most predominant mechanism (76%). Among *qnr* genes, *qnrD*, *qnrA* and *qnrS* genes were detected. *qnrD* was highly reported in isolates of Proteus and Providencia, therefore, it was hypothesized that it originated in family Morganellaceae then disseminated to other Enterobacterales [64]. Previous studies reported detection of *acc (6’)-Ib-cr, qnrD*, and *qnr A* among quinolone resistant proteus isolates [6, 52].

Fosfomycin has attracted a great attention for treating serious systemic infections caused by MDR Enterobacterales. However, resistance to fosfomycin have emerged [65]. Among the tested isolates, (22.7%) were fosfomycin resistant which coincide with recent studies in Brazil and Lebanon [6, 66]. Among the tested isolates, 81.8% were resistant to aminoglycosides. Recent studies in Egypt recorded resistance rate 37.9 to 53.2% [9, 10, 67], while in Ghana, India and Japan, higher rates of resistance (54–100%) were recorded [18, 48, 55, 68]. In addition, 87.9% of the isolates were resistant to trimethoprim/sulfamethoxazole which coincide with recent studies in Egypt [9, 10, 12]. Similarly, elevated rates of resistance were recoded worldwide [3, 18, 52].

Different genetic mechanisms are involved in the acquisition of resistance genes to different antibiotic classes. Horizontal gene transfer, via plasmids, transposons and integrons, is a major cause of the spread of antimicrobial resistance and turn *P. mirabilis* into MDR, XDR or even PDR resistant [11]. Integrons are not self- transferable elements, however they are frequently located on transposons or plasmids, allowing efficient gene transfer. More than 100 gene cassettes bearing resistance to different classes of antibiotics have been reported [51, 52, 69, 70]. Class I and II integrons were detected in 86.4% of the tested isolates. Sequencing analysis of their variable region revealed that they carried distinctive cassettes encoding aminoglycosides and trimethoprim resistance determinants mainly. Previous reports have also indicated that most integrons-carrying genes are coding for aminoglycosides and trimethoprim resistance [52, 69–71].

MDR phenomenon is frequently linked to integrons as they can be transferred, integrated, expressed, and causes distribution of several antimicrobial resistance genes [72].

The high rate of MDR and emergence of XDR among the tested isolates along with their enormous diversity (ERIC-PCR genotyping) could be explained by horizontal transfer of resistance determinants among bacterial isolates in hospitals. Variable rates of MDR (14.5–100%) were reported worldwide among *P. mirabilis* clinical isolates [3, 52, 69]. Previous studies in Egypt reported MDR (29.3–87.2%) among *P. mirabilis* clinical isolates [9, 10, 67]. A recent study reported 22.8% MDR, 31.4% XDR, and 8.5% PDR, which is considered the first report of PDR *P. mirabilis* in Egypt [12].

## Conclusion

The elevated rate of MDR and emergence of XDR among *P. mirabilis* clinical isolates poses a public threat in Egypt limiting the therapeutic options for management of infections caused by these superbugs. Appropriate use of antimicrobial agents in the health setting along with surveillance of antimicrobial resistance profiles and the underlying resistance determinants are highly requested for controlling the spread of antimicrobial resistance and emergence of PDR stains in the future.

### Electronic supplementary material

Below is the link to the electronic supplementary material.

Supplementary Material 1

Supplementary Material 2

Supplementary Material 3

Supplementary Material 4

Supplementary Material 5

## Data Availability

All data generated or analyzed during this study are included in this published article and its supplementary information files.

## Abbreviations

MDR: Multidrug-resistant
XDR: Extensive drug resistant
PDR: Pandrug-resistant
ESBLs: Extended spectrum β-lactamases
AmpCs: Plasmid mediated cephalosporinase
MBLs: Metallo β‑lactamases
ERIC-PCR: Enterobacterial repetitive intergenic consensus PCR
MCA: Multiple correspondence analysis
PMQR: Plasmid mediated quinolone resistance
