# Supplementary material for: Emergence of extensive drug resistance and high prevalence of multidrug resistance among clinical Proteus mirabilis isolates in Egypt
Source: Ann Clin Microbiol Antimicrob. 2024 May 24;23:46. doi: 10.1186/s12941-024-00705-3 (PMC11127457; doi:10.1186/s12941-024-00705-3)
Supplement: Supplementary file 1 — Supplementary Material 1 [file 12941_2024_705_MOESM1_ESM.docx]

**Table S1:** Characterization and distribution of antimicrobial resistance, resistance determinants, and class I and II integrons among the tested *P. mirabilis* clinical isolates.

| **Isolate No.** | **Isolation source** | **Sex** | **Resistance profile** | **Detection of β-Lactamases** | | | | | | **PMQR** | **Class I Integrons** | | **Class II Integrons** | | **Resistance Pattern** | **RS** |
| --- | --- | --- | --- | --- | --- | --- | --- | --- | --- | --- | --- | --- | --- | --- | --- | --- |
|  |  |  |  | **Acidmetric** | **ESBLs** | **AmpCs** | **MBLs** | **Carb.s** | **β-lactamases**  **encoding genes** |  | ***IntI1*** | **Gene Cassette** | ***IntI2*** | **Gene Cassette** |  |  |
| **Pm01** | Urine | M | AM, PRL, AMC, SAM, CZ, CXM, CTX, CAZ, MA, CFP, FEP, FOX, NA, NOR, OFX, CIP, LOM, LEV, S, K, DO, MI, SXT, C, FOS, F | **+** | **+** | **+** | **-** | **-** | *bla*_TEM_*, bla_CIT-M_* | *acc (6')-Ib-cr* | **+** | *aadB-aadA2, dfrA7* | **+** | *estXVr-sat2-aadA1* | MDR | 14 |
| **Pm02** | T-Tube drain | F | AM, PRL, AMC, SAM, CZ, CXM, CTX, CAZ, MA, CFP, FEP, FOX, NA, OFX, CIP, LOM, LEV, GAT, S, K, CN, NET, TOB, DO, MI, SXT, C, FOS, F | **+** | **+** | **+** | **-** | **-** | *bla*_TEM_*, bla*_OXA-1-like_*,*  *bla_CIT-M_* | *acc (6')-Ib-cr, qnrA, qnrD* | **+** | *aadA2-lnuF* | **+** | *lnuF-dfrA1-aadA1* | MDR | 14 |
| **Pm03** | T-Tube drain | M | AM, PRL, AMC, SAM, TPZ, CZ, CXM, MA, CFP, FEP, NA, NOR, OFX, CIP, LOM, LEV, GAT, S, K, CN, NET, TOB, AK, DO, MI, SXT, C, FOS, F | **-** | **-** | **-** | **-** | **-** | - | *acc (6')-Ib-cr, qnrA, qnrD* | **+** | *dfrA17-aadA5* | **+** | *dfrA1-sat1-aadA1* | MDR | 14 |
| **Pm04** | Urine | M | AM, PRL, AMC, CZ, FEP, DO, MI, F | **-** | **-** | **-** | **-** | **-** | - | - | **+** | *dfrA7* | **+** | NA | MDR | 6 |
| **Pm05** | Urine | M | AM, PRL, AMC, CZ, FEP, DO, MI, F | **-** | **-** | **-** | **-** | **-** | - | - | **+** | *dfrA7* | **+** | NS | MDR | 6 |
| **Pm06** | Urine | F | AM, AMC, CZ, FEP, DO, MI, F | **-** | **-** | **-** | **-** | **-** | - | - | **+** | NA | **+** | NS | MDR | 6 |
| **Pm07** | Blood | F | AM, PRL, AMC, SAM, TPZ, CZ, CXM, CTX, CRO, MA, CFP, FEP, AZT, NA, NOR, OFX, CIP, LOM, LEV, GAT, S, K, CN, NET, TOB, AK, DO, MI, SXT, C, FOS, F | **+** | **+** | **-** | **-** | **-** | *bla*_TEM_*, bla_CTX-M_,*  *bla*_OXA-1-like_ | *acc (6')-Ib-cr* | **+** | *dfrA17-aadA5* | **+** | *dfrA1-sat1-aadA1* | XDR | 15 |
| **Pm08** | Sputum | F | AM, PRL, AMC, SAM, TPZ, CZ, MA, FEP, NA, NOR, OFX, CIP, LOM, LEV, GAT, S, K, CN, NET, TOB, AK, DO, MI, SXT, C, FOS, F | **-** | **-** | **-** | **-** | **-** | - | *acc (6')-Ib-cr, qnrA, qnrD* | **+** | NA | **+** | *dfrA1-sat1-aadA1* | MDR | 13 |
| **Pm09** | Burn swab | M | AM, PRL, AMC, SAM, CZ, CXM, CTX, CRO, MA, CFP, FEP, AZT, NA, NOR, OFX, CIP, LOM, LEV, GAT, S, K, CN, NET, TOB, AK, DO, MI, SXT, C, FOS, F | **+** | **+** | **-** | **-** | **-** | *bla*_SHV_*, bla*_TEM_*,*  *bla_CTX-M_, bla*_OXA-1-like_ | *acc (6')-Ib-cr, qnrA, qnrD* | **+** | NA | **+** | *dfrA1-sat1-aadA1* | MDR | 14 |
| **Pm10** | Urine | M | AM, PRL, AMC, CZ, FEP, DO, MI, F | **-** | **-** | **-** | **-** | **-** | - | - | **+** | NA | **+** | NS | MDR | 6 |
| **Pm11** | Urine | M | AM, PRL, AMC, SAM, CZ, FEP, S, K, DO, MI, SXT, C, F | **-** | **-** | **-** | **-** | **-** | - | - | **+** | NA | **+** | *dfrA1-sat2* | MDR | 9 |
| **Pm12** | Burn swab | F | AM, PRL, AMC, CZ, FEP, DO, MI, C, F | **-** | **-** | **-** | **-** | **-** | - | - | **-** | - | **-** | - | MDR | 7 |
| **Pm13** | Burn swab | F | AM, PRL, AMC, CZ, CXM, CTX, CRO, CAZ, MA, CFP, FEP, AZT, NA, NOR, OFX, CIP, LOM, LEV, GAT, S, K, CN, NET, TOB, AK, DO, MI, SXT, C, F | **+** | **+** | **-** | **-** | **-** | *bla*_SHV_*, bla*_TEM_*,*  *bla*_OXA-1-like_ | *acc (6')-Ib-cr, qnrA, qnrS, qnrD* | **+** | NS | **+** | *dfrA1-sat1-aadA1* | MDR | 13 |
| **Pm14** | Sputum | F | AM, PRL, AMC, SAM, CZ, CXM, CTX, CRO, CAZ, MA, CFP, FEP, AZT, NA, NOR, OFX, CIP, LOM, LEV, GAT, S, K, CN, NET, TOB, AK, DO, MI, SXT, C, F | **+** | **+** | **-** | **-** | **-** | *bla*_TEM_*, bla*_OXA-1-like_ | *acc (6')-Ib-cr, qnrA, qnrD* | **+** | NS | **+** | *dfrA1-sat1-aadA1* | MDR | 13 |
| **Pm15** | Urine | M | AM, PRL, AMC, SAM, CZ, CXM, CTX, CAZ, MA, CFP, FEP, S, K, CN, NET, TOB, DO, MI, SXT, C, F | **+** | **+** | **-** | **-** | **-** | *bla*_TEM_*, bla*_OXA-1-like_ | - | **+** | NA | **+** | NS | MDR | 11 |
| **Pm16** | T-Tube drain | F | AM, PRL, AMC, SAM, CZ, CXM, CTX, CRO, MA, CFP, FEP, AZT, NA, NOR, OFX, CIP, LOM, LEV, GAT, S, K, NET, TOB, DO, MI, SXT, C, FOS, F | **+** | **+** | **-** | **-** | **-** | *bla*_SHV_*, bla*_TEM_*,*  *bla_CTX-M_, bla*_OXA-1-like_ | *acc (6')-Ib-cr, qnrD* | **+** | NA | **+** | *dfrA1-sat1-aadA1* | MDR | 14 |
| **Pm17** | Sputum | F | AM, PRL, AMC, SAM, CZ, CTX, CRO, CAZ, MA, CFP, FEP, FOX, NA, CIP, LOM, S, K, DO, MI, SXT, C, F | **+** | **+** | **+** | **-** | **-** | *bla*_TEM_*, bla*_OXA-1-like_*,*  *bla_AmpC_, bla_CIT-M_* | - | **+** | *dfrA7* | **+** | *estXVr-sat2-aadA1* | MDR | 12 |
| **Pm18** | Sputum | F | AM, PRL, AMC, CZ, FEP, DO, MI, SXT, C, F | **-** | **-** | **-** | **-** | **-** | - | - | **+** | *dfrA7* | **-** | - | MDR | 8 |
| **Pm19** | Sputum | M | AM, PRL, AMC, CZ, CXM, CTX, CAZ, MA, FEP, NA, CIP, LOM, LEV, S, K, DO, MI, SXT, C, F | **+** | **+** | **-** | **-** | **-** | *bla*_TEM_*, bla*_OXA-1-like_ | *qnrD* | **+** | NA | **+** | NS | MDR | 12 |
| **Pm20** | Burn swab | F | AM, PRL, AMC, SAM, CZ, CTX, CRO, CAZ, MA, FEP, FOX, NA, CIP, LOM, S, K, DO, MI, SXT, C, F | **+** | **+** | **+** | **-** | **-** | *bla*_TEM_*, bla*_OXA-1-like_*,*  *bla_CIT-M_* | - | **+** | *aadB-aadA2, dfrA7* | **+** | *lnuF-dfrA1-aadA1* | MDR | 12 |
| **Pm21** | Urine | F | AM, PRL, AMC, SAM, CZ, CXM, CTX, CAZ, MA, FEP, FOX, NA, NOR, OFX, CIP, LOM, LEV, GAT, S, K, CN, NET, DO, MI, SXT, C, F | **+** | **+** | **+** | **-** | **-** | *bla*_SHV_*, bla*_TEM_*,*  *bla*_OXA-1-like_ | *acc (6')-Ib-cr, qnrA, qnrS, qnrD* | **+** | *aadA1* | **+** | *lnuF-dfrA1-aadA1* | MDR | 13 |
| **Pm22** | Urine | F | AM, PRL, AMC, CZ, CXM, CTX, CRO, MA, CFP, FEP, AZT, NA, NOR, OFX, CIP, LOM, LEV, GAT, S, K, NET, DO, MI, SXT, C, FOS, F | **+** | **+** | **-** | **-** | **-** | *bla*_TEM_*, bla_CTX-M_,*  *bla*_OXA-1-like_ | - | **-** | - | **+** | *dfrA1-sat1-aadA1* | MDR | 14 |
| **Pm23** | Blood | M | AM, PRL, AMC, CZ, CXM, CTX, CRO, MA, CFP, FEP, AZT, NA, NOR, OFX, CIP, LOM, LEV, GAT, S, K, TOB, DO, MI, SXT, C, FOS, F | **+** | **+** | **-** | **-** | **-** | *bla*_TEM_*, bla_CTX-M_,*  *bla*_OXA-1-like_ | - | **+** | NA | **+** | *dfrA1-sat1-aadA1* | MDR | 14 |
| **Pm24** | T-Tube drain | F | AM, PRL, AMC, CZ, FEP, NA, CIP, LOM, LEV, S, K, DO, MI, SXT, C, F | **-** | **-** | **-** | **-** | **-** | - | *qnrD* | **-** | - | **+** | *dfrA1-sat1-aadA1* | MDR | 10 |
| **Pm25** | Urine | F | AM, PRL, AMC, CZ, FEP, NA, CIP, LOM, LEV, S, K, DO, MI, SXT, C, F | **-** | **-** | **-** | **-** | **-** | - | *acc (6')-Ib-cr* | **+** | NA | **+** | *dfrA1-sat1-aadA1* | MDR | 10 |
| **Pm26** | T-Tube drain | F | AM, PRL, AMC, CZ, CXM, CTX, CRO, MA, CFP, FEP, AZT, NA, NOR, OFX, CIP, LOM, LEV, GAT, S, K, NET, DO, MI, SXT, C, FOS, F | **+** | **+** | **-** | **-** | **-** | *bla_CTX-M_* | *acc (6')-Ib-cr, qnrD* | **+** | NA | **+** | *dfrA1-sat1-aadA1* | MDR | 14 |
| **Pm27** | T-Tube drain | F | AM, PRL, AMC, CZ, FEP, NA, CIP, LOM, LEV, GAT, S, K, DO, MI, SXT, C, F | **-** | **-** | **-** | **-** | **-** | - | *qnrD* | **-** | - | **+** | *dfrA1-sat1-aadA1* | MDR | 10 |
| **Pm28** | Blood | F | AM, PRL, AMC, SAM, CZ, CXM, CTX, CAZ, MA, FEP, S, K, CN, DO, MI, SXT, C, F | **+** | **+** | **-** | **-** | **-** | *bla*_SHV_*, bla*_TEM_*,*  *bla*_OXA-1-like_ | - | **+** | NA | **+** | NA | MDR | 11 |
| **Pm29** | Burn swab | F | AM, PRL, AMC, SAM, CZ, CXM, CTX, CRO, MA, CFP, FEP, AZT, NA, NOR, OFX, CIP, LOM, LEV, GAT, S, K, CN, NET, TOB, DO, MI, SXT, C, FOS, F | **+** | **+** | **-** | **-** | **-** | *bla*_TEM_*, bla_CTX-M_,*  *bla*_OXA-1-like_ | *acc (6')-Ib-cr, qnrA, qnrD* | **+** | *dfrA17-aadA5* | **+** | *dfrA1-sat1-aadA1* | MDR | 14 |
| **Pm30** | Diabetic foot lesion | F | AM, PRL, AMC, SAM, CZ, FEP, NA, OFX, CIP, LOM, LEV, S, K, CN, NET, TOB, DO, MI, SXT, C, F | **-** | **-** | **-** | **-** | **-** | - | *acc (6')-Ib-cr* | **+** | *dfrA14-arr-3-bla*_OXA-10_-*aadA15, aadA1* | **+** | NS | MDR | 10 |
| **Pm31** | Diabetic foot lesion | F | AM, PRL, AMC, SAM, CZ, CXM, CTX, CRO, CAZ, MA, CFP, FEP, AZT, NA, NOR, OFX, CIP, LOM, LEV, GAT, S, K, DO, MI, SXT, C, FOS, F | **+** | **+** | **-** | **-** | **-** | *bla*_TEM_*, bla_CTX-M_,*  *bla*_OXA-1-like_ | *acc (6')-Ib-cr, qnrA* | **+** | *dfrA14-arr-3-bla*_OXA-10_-*aadA15* | **+** | NS | MDR | 14 |
| **Pm32** | Diabetic foot lesion | F | AM, PRL, AMC, CZ, FEP, NA, NOR, OFX, CIP, LOM, LEV, GAT, S, K, CN, DO, MI, SXT, C, FOS, F | **-** | **-** | **-** | **-** | **-** | - | *qnrD* | **+** | *dfrA17-aadA5* | **+** | NS | MDR | 11 |
| **Pm33** | Diabetic foot lesion | F | AM, PRL, AMC, SAM, TPZ, CZ, MA, FEP, NA, OFX, CIP, LOM, LEV, GAT, S, K, CN, AK, DO, MI, SXT, C, F | **-** | **-** | **-** | **-** | **-** | - | *acc (6')-Ib-cr* | **+** | *aadB-aadA2* | **+** | *estXVr-sat2-aadA1* | MDR | 12 |
| **Pm34** | Diabetic foot lesion | M | AM, PRL, AMC, SAM, CZ, CXM, CTX, CRO, CAZ, MA, CFP, FEP, FOX, IPM, ETP, DOR, NA, NOR, OFX, CIP, LOM, LEV, GAT, S, K, CN, NET, TOB, DO, MI, SXT, C, F | **+** | **+** | **+** | **+** | **+** | *bla*_NDM-1_*,*  *bla*_OXA-48-like_ | *qnrA* | **+** | *aadA1* | **+** | *dfrA1-sat1-aadA1* | MDR | 14 |
| **Pm35** | Diabetic foot lesion | M | AM, PRL, AMC, CZ, FEP, DO, MI, F | **-** | **-** | **-** | **-** | **-** | - | - | **-** | - | **-** | - | MDR | 6 |
| **Pm36** | Thigh boils swab | F | AM, PRL, AMC, SAM, CZ, CXM, CTX, CRO, CAZ, MA, CFP, FEP, AZT, NA, NOR, OFX, CIP, LOM, LEV, GAT, S, K, CN, NET, TOB, AK, DO, MI, SXT, C, F | **+** | **+** | **-** | **-** | **-** | *bla*_TEM_*, bla*_OXA-1-like_ | *acc (6')-Ib-cr, qnrA* | **+** | NS | **+** | *dfrA1-sat1-aadA1* | MDR | 13 |
| **Pm37** | Bedsore swab | F | AM, PRL, AMC, CZ, FEP, NA, OFX, CIP, LOM, LEV, GAT, S, K, CN, NET, TOB, AK, DO, MI, SXT, C, F | **-** | **-** | **-** | **-** | **-** | - | *qnrA* | **+** | NA | **+** | *dfrA1-sat1-aadA1* | MDR | 10 |
| **Pm38** | Diabetic foot lesion | F | AM, PRL, AMC, SAM, CZ, CXM, CTX, CRO, MA, CFP, FEP, AZT, NA, NOR, OFX, CIP, LOM, LEV, GAT, S, K, CN, NET, TOB, DO, MI, SXT, C, F | **+** | **+** | **-** | **-** | **-** | *bla*_TEM_*, bla_CTX-M_,*  *bla*_OXA-1-like_ | *-* | **+** | *aadA2-lnuF* | **+** | NS | MDR | 13 |
| **Pm39** | Diabetic foot lesion | M | AM, PRL, AMC, SAM, CZ, CXM, CTX, CRO, CAZ, MA, CFP, FEP, AZT, NA, NOR, OFX, CIP, LOM, LEV, GAT, S, K, CN, NET, TOB, AK, DO, MI, SXT, C, F | **+** | **+** | **-** | **-** | **-** | *bla*_TEM_*, bla*_OXA-1-like_ | *acc (6')-Ib-cr, qnrD* | **+** | *dfrA17-aadA5* | **-** | - | MDR | 13 |
| **Pm40** | Urine | M | AM, PRL, AMC, SAM, CZ, CXM, CTX, CRO, CAZ, MA, CFP, FEP, FOX, IPM, ETP, DOR, NA, NOR, OFX, CIP, LOM, LEV, GAT, S, K, CN, TOB, DO, MI, SXT, C, FOS, F | **+** | **+** | **+** | **+** | **+** | *bla*_SHV_*, bla*_TEM_*,*  *bla*_OXA-1-like_*, bla*_OXA-48-like_ | *acc (6')-Ib-cr, qnrA* | **+** | NA | **+** | *dfrA1-sat1-aadA1* | XDR | 15 |
| **Pm41** | Urine | F | AM, PRL, AMC, SAM, CZ, CXM, CTX, CRO, MA, CFP, FEP, S, K, DO, MI, SXT, C, F | **+** | **+** | **-** | **-** | **-** | *bla*_TEM_*, bla_CTX-M_,*  *bla*_OXA-1-like_ | - | **+** | *dfrA7* | **+** | NS | MDR | 11 |
| **Pm42** | Urine | F | AM, AMC, CZ, FEP, DO, MI, SXT, C, F | **-** | **-** | **-** | **-** | **-** | - | - | **-** | - | **-** | - | MDR | 8 |
| **Pm43** | Urine | M | AM, AMC, CZ, FEP, DO, MI, SXT, F | **-** | **-** | **-** | **-** | **-** | - | - | **-** | - | **-** | - | MDR | 7 |
| **Pm44** | Urine | M | AM, PRL, AMC, SAM, TPZ, CZ, CXM, CTX, CRO, CAZ, MA, CFP, FEP, FOX, IPM, MEM, ETP, DOR, AZT, NA, NOR, OFX, CIP, LOM, LEV, GAT, S, K, CN, NET, TOB, AK, DO, MI, SXT, C, F | **+** | **+** | **+** | **+** | **+** | *bla*_SHV_*, bla*_OXA-1-like_*,*  *bla*_NDM-1_*, bla*_OXA-48-like_ | *acc (6')-Ib-cr, qnrA, qnrS, qnrD* | **+** | *dfrA17-aadA5*, *dfrA7* | **+** | *dfrA1-sat1-aadA1* | XDR | 16 |
| **Pm45** | Urine | F | AM, PRL, AMC, SAM, CZ, CXM, CTX, CRO, CAZ, MA, CFP, FEP, AZT, NA, OFX, CIP, LOM, LEV, GAT, S, K, CN, NET, TOB, AK, DO, MI, SXT, C, F | **+** | **+** | **-** | **-** | **-** | *bla*_TEM_*, bla*_OXA-1-like_ | *acc (6')-Ib-cr* | **+** | NS | **+** | *dfrA1-sat1-aadA1* | MDR | 13 |
| **Pm46** | Urine | M | AM, PRL, AMC, CZ, CXM, CTX, CRO, CAZ, MA, CFP, FEP, AZT, NA, NOR, OFX, CIP, LOM, LEV, GAT, S, K, NET, TOB, AK, DO, MI, SXT, C, F | **+** | **+** | **-** | **-** | **-** | *bla*_TEM_*, bla*_OXA-1-like_ | *acc (6')-Ib-cr, qnrD* | **+** | NA | **+** | NA | MDR | 13 |
| **Pm47** | Urine | F | AM, PRL, AMC, SAM, TPZ, CZ, CXM, CTX, CRO, CAZ, MA, CFP, FEP, FOX, IPM, MEM, ETP, DOR, NA, NOR, OFX, CIP, LOM, LEV, GAT, S, K, CN, NET, TOB, AK, DO, MI, SXT, C, F | **+** | **+** | **+** | **+** | **+** | *bla*_TEM_*, bla*_OXA-1-like_*, bla*_AmpC_*, bla*_NDM-1_*,*  *bla*_OXA-48-like_ | *acc (6')-Ib-cr, qnrA, qnrS, qnrD* | **+** | *dfrA17-aadA5*, *dfrA7* | **+** | *dfrA1-sat1-aadA1* | XDR | 15 |
| **Pm48** | Urine | M | AM, AMC, CZ, FEP, NA, CIP, LOM, DO, MI, SXT, C, F | **-** | **-** | **-** | **-** | **-** | - | *acc (6')-Ib-cr, qnrA, qnrD* | **+** | *dfrA17-aadA5*, *dfrA7* | **+** | *dfrA1-sat1-aadA1* | MDR | 9 |
| **Pm49** | Urine | F | AM, PRL, AMC, SAM, CZ, CXM, MA, FEP, NA, CIP, LOM, LEV, S, K, CN, TOB, AK, DO, MI, SXT, F | **-** | **-** | **-** | **-** | **-** | - | *acc (6')-Ib-cr* | **+** | *aadB-aadA2* | **+** | *dfrA1-sat1-aadA1* | MDR | 11 |
| **Pm50** | Urine | F | AM, PRL, AMC, SAM, CZ, CXM, CTX, CRO, CAZ, MA, CFP, FEP, AZT, NA, NOR, OFX, CIP, LOM, LEV, GAT, S, K, CN, NET, TOB, AK, DO, MI, SXT, C, F | **+** | **+** | **-** | **-** | **-** | *bla*_TEM_*, bla*_OXA-1-like_ | *acc (6')-Ib-cr, qnrA, qnrD* | **+** | *dfrA17-aadA5* | **+** | *dfrA1-sat1-aadA1* | MDR | 13 |
| **Pm51** | Diabetic foot lesion | M | AM, PRL, AMC, SAM, CZ, CXM, CTX, CRO, CAZ, MA, CFP, FEP, AZT, NA, NOR, OFX, CIP, LOM, LEV, GAT, S, K, CN, NET, TOB, AK, DO, MI, SXT, C, F | **+** | **+** | **-** | **-** | **-** | *bla*_TEM_*, bla*_OXA-1-like_ | *acc (6')-Ib-cr* | **+** | NS | **+** | *dfrA1-sat1-aadA1* | MDR | 13 |
| **Pm52** | Sputum | F | AM, PRL, AMC, CZ, FEP, NA, OFX, CIP, LOM, LEV, S, K, CN, NET, TOB, AK, DO, MI, SXT, C, F | **-** | **-** | **-** | **-** | **-** | - | *acc (6')-Ib-cr, qnrD* | **-** | - | **-** | - | MDR | 10 |
| **Pm53** | Wound swab | F | AM, PRL, AMC, SAM, TPZ, CZ, CXM, CTX, CRO, CAZ, MA, CFP, FEP, FOX, IPM, MEM, ETP, DOR, AZT, NA, NOR, OFX, CIP, LOM, LEV, GAT, S, K, CN, NET, TOB, AK, DO, MI, SXT, F | **+** | **+** | **+** | **+** | **+** | *bla*_TEM_*, bla*_OXA-1-like_*,*  *bla*_NDM-1_*, bla*_OXA-48-like_ | *acc (6')-Ib-cr, qnrA, qnrS, qnrD* | **+** | *dfrA7* | **+** | *dfrA1-sat1-aadA1* | XDR | 15 |
| **Pm54** | Sputum | M | AM, PRL, AMC, CZ, MA, FEP, NA, NOR, OFX, CIP, LOM, LEV, GAT, S, K, CN, NET, TOB, AK, DO, MI, SXT, C, F | **-** | **-** | **-** | **-** | **-** | - | *acc (6')-Ib-cr* | **+** | NS | **+** | *dfrA1-sat1-aadA1* | MDR | 11 |
| **Pm55** | Wound swab | M | AM, PRL, AMC, SAM, CZ, FEP, NA, NOR, OFX, CIP, LOM, LEV, GAT, S, K, CN, NET, TOB, AK, DO, MI, SXT, C, F | **-** | **-** | **-** | **-** | **-** | - | *acc (6')-Ib-cr* | **+** | NS | **+** | *dfrA1-sat1-aadA1* | MDR | 10 |
| **Pm56** | Diabetic foot lesion | F | AM, PRL, AMC, CZ, CXM, CTX, CRO, CAZ, MA, CFP, FEP, AZT, NA, OFX, CIP, LOM, LEV, S, K, CN, NET, TOB, AK, DO, MI, SXT, C, F | **+** | **+** | **-** | **-** | **-** | *bla*_SHV_*, bla*_TEM_*,*  *bla*_OXA-1-like_ | *acc (6')-Ib-cr* | **+** | NS | **+** | *dfrA1-sat1-aadA1* | MDR | 13 |
| **Pm57** | Diabetic foot lesion | M | AM, PRL, AMC, SAM, CZ, CXM, CTX, CRO, CAZ, MA, CFP, FEP, FOX, AZT, NA, NOR, OFX, CIP, LOM, LEV, GAT, S, K, CN, NET, TOB, AK, DO, MI, SXT, C, F | **+** | **+** | **+** | **-** | **-** | *bla*_TEM_*, bla_CTX-M_,*  *bla*_OXA-1-like_ | *acc (6')-Ib-cr, qnrD* | **+** | *aadA2-lnuF* | **+** | NS | MDR | 14 |
| **Pm58** | Wound swab | M | AM, PRL, AMC, CZ, CFP, FEP, S, K, NET, AK, DO, MI, C, F | **-** | **-** | **-** | **-** | **-** | - | - | **+** | NS | **+** | *dfrA1-sat1-aadA1* | MDR | 9 |
| **Pm59** | Aspirate swab | F | AM, PRL, AMC, SAM, CZ, CXM, CTX, CRO, CAZ, MA, CFP, FEP, FOX, AZT, NA, NOR, OFX, CIP, LOM, LEV, GAT, S, K, CN, NET, TOB, AK, DO, MI, SXT, C, F | **+** | **+** | **+** | **-** | **-** | *bla*_TEM_*, bla*_OXA-1-like_*,*  *bla_CIT-M_* | *acc (6')-Ib-cr, qnrD* | **+** | *dfrA17-aadA5* | **+** | *dfrA1-sat1-aadA1* | MDR | 14 |
| **Pm60** | Femoral wound swab | F | AM, PRL, AMC, CZ, CXM, CTX, CRO, CAZ, MA, CFP, FEP, AZT, NA, NOR, OFX, CIP, LOM, LEV, GAT, S, K, CN, NET, TOB, AK, DO, MI, SXT, C, F | **+** | **+** | **-** | **-** | **-** | *bla*_TEM_*, bla*_OXA-1-like_ | *acc (6')-Ib-cr, qnrA* | **+** | NS | **+** | *dfrA1-sat1-aadA1* | MDR | 13 |
| **Pm61** | Urine | M | AM, PRL, AMC, CZ, MA, FEP, DO, MI, C, F | **-** | **-** | **-** | **-** | **-** | - | - | **+** | NS | **-** | - | MDR | 8 |
| **Pm62** | Urine | F | AM, PRL, AMC, CZ, FEP, DO, MI, SXT, C, F | **-** | **-** | **-** | **-** | **-** | - | - | **-** | - | **-** | - | MDR | 8 |
| **Pm63** | Diabetic foot lesion | F | AM, PRL, AMC, SAM, CZ, CXM, CTX, CRO, CAZ, MA, CFP, FEP, FOX, IPM, MEM, ETP, DOR, NA, NOR, OFX, CIP, LOM, LEV, GAT, S, K, NET, TOB, AK, DO, MI, SXT, C, F | **+** | **+** | **+** | **-** | **+** | *bla*_TEM_*, bla*_OXA-1-like_*,*  *bla*_OXA-48-like_ | *qnrA, qnrD* | **+** | *aadA2-lnuF, aadA1* | **+** | *dfrA1-sat1-aadA1* | MDR | 14 |
| **Pm64** | Bedsore swab | F | AM, PRL, AMC, SAM, TPZ, CZ, CXM, CTX, CRO, CAZ, MA, CFP, FEP, FOX, IPM, MEM, ETP, DOR, AZT, NA, NOR, OFX, CIP, LOM, LEV, GAT, S, K, CN, NET, TOB, AK, DO, MI, SXT, C, F | **+** | **+** | **+** | **+** | **+** | *bla*_OXA-1-like_*, bla*_NDM-1_*, bla*_OXA-48-like_ | *acc (6')-Ib-cr, qnrA, qnrS* | **+** | *dfrA17-aadA5*, *dfrA7* | **+** | *dfrA1-sat1-aadA1* | XDR | 16 |
| **Pm65** | Thigh boils swab | F | AM, PRL, AMC, SAM, TPZ, CZ, MA, FEP, NA, NOR, OFX, CIP, LOM, LEV, GAT, S, K, AK, DO, MI, SXT, C, F | **-** | **-** | **-** | **-** | **-** | - | *acc (6')-Ib-cr, qnrA, qnrD* | **+** | NS | **+** | NS | MDR | 12 |
| **Pm66** | Urine | F | AM, PRL, AMC, SAM, CZ, CXM, CTX, CRO, CAZ, MA, CFP, FEP, AZT, NA, NOR, OFX, CIP, LOM, LEV, GAT, S, K, AK, DO, MI, SXT, C, FOS, F | **+** | **+** | **-** | **-** | **-** | *bla*_TEM_*, bla_CTX-M_,*  *bla*_OXA-1-like_ | *acc (6')-Ib-cr, qnrA* | **+** | *dfrA14-arr-3-bla*_OXA-10_-*aadA15* | **+** | NS | MDR | 14 |

NA: Not amplified, NS: Not Sequenced (<800bp), MDR: Multi Drug Resistant, XDR: Extensive Drug Resistant, RS: Resistance ScoreSex; F:Female, M:Male

AM, Ampicillin; PRL, Piperacillin; AMC, Amoxicillin-Clavulanic acid; SAM, Ampicillin-Sulbactam; TPZ, Piperacillin -Tazobactam; CZ, Cefazolin; CXM, Cefuroxime; CTX, Cefotaxime; CRO, Ceftriaxone; CAZ, Ceftazidime; MA, Cefamandole; CFP, Cefoperazone; FEP, Cefepime; FOX, Cefoxitin; IPM, Imipenem; MEM, Meropenem; ETP, Ertapenem; DOR, Doripenem; AZT, Aztreonam; NA, Nalidixic acid; NOR, Norfloxacin; OFX, Ofloxacin; CIP, Ciprofloxacin; LOM, Lomefloxacin; LEV, Levofloxacin; GAT, Gatifloxacin; S, Streptomycin; K, Kanamycin; CN, Gentamicin; NET, Netilmicin; TOB, Tobramycin; AK, Amikacin; DO, Doxycycline; MI, Minocycline; SXT, Trimethoprim-Sulfamethoxazole; C, Chloramphenicol; FOS, Fosfomycin; F, Nitrofurantoin.
