# Supplementary figures and images for: Emergence of extensive drug resistance and high prevalence of multidrug resistance among clinical Proteus mirabilis isolates in Egypt
Source: Ann Clin Microbiol Antimicrob. 2024 May 24;23:46. doi: 10.1186/s12941-024-00705-3 (PMC11127457; doi:10.1186/s12941-024-00705-3)

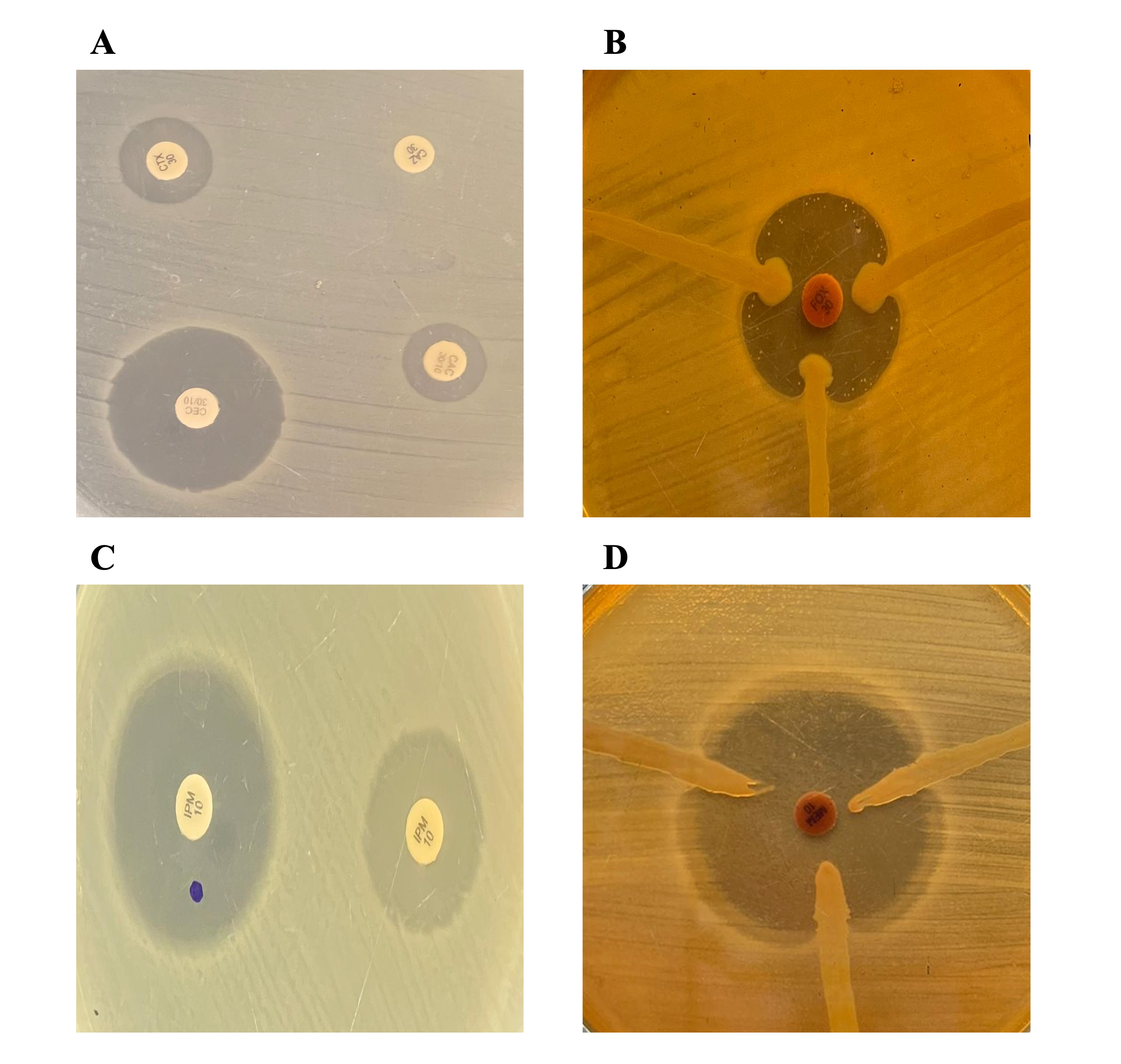

Supplement: Supplementary file 2 — Supplementary Material 2 [file 12941_2024_705_MOESM2_ESM.png]

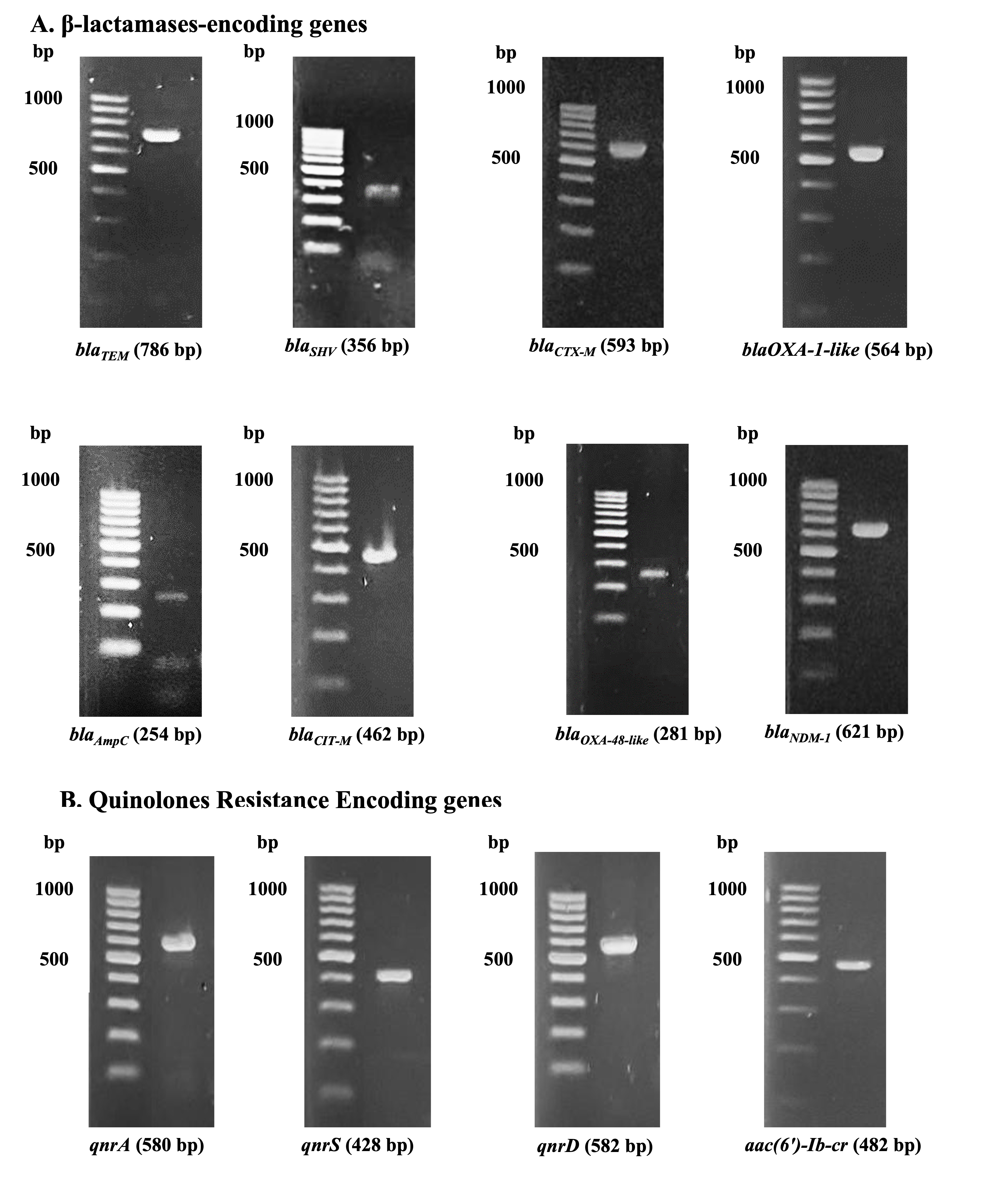

Supplement: Supplementary file 3 — Supplementary Material 3 [file 12941_2024_705_MOESM3_ESM.png]

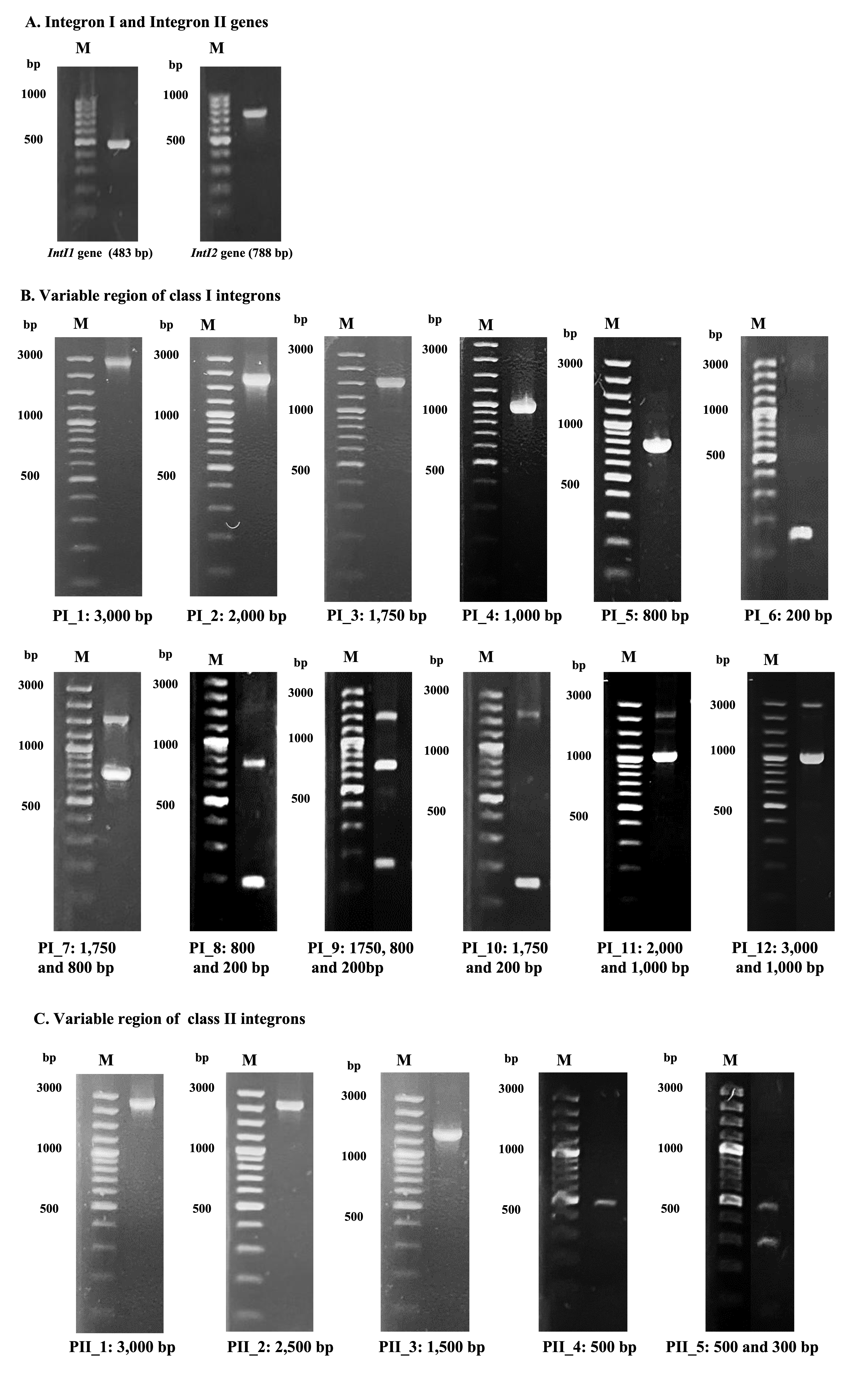

Supplement: Supplementary file 4 — Supplementary Material 4 [file 12941_2024_705_MOESM4_ESM.png]
