# Supplementary material for: Emergence of extensive drug resistance and high prevalence of multidrug resistance among clinical Proteus mirabilis isolates in Egypt
Source: Ann Clin Microbiol Antimicrob. 2024 May 24;23:46. doi: 10.1186/s12941-024-00705-3 (PMC11127457; doi:10.1186/s12941-024-00705-3)
Supplement: Supplementary file 5 — Supplementary Material 5 [file 12941_2024_705_MOESM5_ESM.docx]

**Table S2: Prevalence of antibiotic resistance among *P. mirabilis* isolated from different clinical sources.**

| **Antimicrobial classes** | **Antimicrobial Agent** | **No. of isolates (%)** | | |
| --- | --- | --- | --- | --- |
|  |  | **Resistant** | **Intermediate** | **Sensitive** |
| Penicillins | Ampicillin | 64 (97) | 2 (3) | 0 (0) |
|  | Piperacillin | 55 (83.3) | 7 (10.6) | 4 (6.1) |
| Penicillins / β-lactamase inhibitors | Ampicillin/Sulbactam | 26 (39.4) | 12 (18.2) | 28 (42.4) |
|  | Amoxicillin/Clavulanic acid | 63 (95.5) | 3 (4.5) | 0 (0) |
| Antipseudomonal penicillins / β- lactamase inhibitors | Piperacillin/Tazobactam | 3 (4.5) | 6 (9.1) | 57 (86.4) |
| First generation cephalosporins | Cefazolin | 66 (100) | 0 (0) | 0 (0) |
| Second generation cephalosporins | Cefuroxime | 32 (48.5) | 6 (9.1) | 28 (42.4) |
| Third generation cephalosporins | Cefotaxime | 38 (57.6) | 0 (0) | 28 (42.4) |
|  | Ceftriaxone | 27 (40.9) | 5 (7.6) | 34 (51.5) |
|  | Ceftazidime | 28 (42.4) | 1 (1.5) | 37 (56.1) |
|  | Cefamandole | 41 (62.1) | 4 (6.1) | 21 (31.8) |
|  | Cefoperazone | 26 (39.4) | 10 (15.1) | 30 (45.5) |
| Fourth generation cephalosporins | Cefepime | 66 (100) | 0 (0) | 0 (0) |
| Cephamycins | Cefoxitin | 6 (9.1) | 8 (12.1) | 52 (78.8) |
| Carbapenems | Ertapenem | 4 (6.1) | 3 (4.5) | 59 (89.4) |
|  | Imipenem | 4 (6.1) | 3 (4.5) | 59 (89.4) |
|  | Meropenem | 4 (6.1) | 1 (1.5) | 61 (92.4) |
|  | Doripenem | 7 (10.6) | 0 (0) | 59 (89.4) |
| Monobactams | Aztreonam | 21 (31.8) | 4 (6.1) | 41 (62.1) |
| Fluoroquinolones | Nalidixic acid | 50 (75.8) | 0 (0) | 16 (24.2) |
|  | Ciprofloxacin | 50 (75.8) | 0 (0) | 16 (24.2) |
|  | Ofloxacin | 34 (51.5) | 8 (12.1) | 24 (36.4) |
|  | Norfloxacin | 20 (30.3) | 15 (22.7) | 31 (47) |
|  | Lomefloxacin | 49 (74.2) | 1 (1.5) | 16 (24.3) |
|  | Levofloxacin | 35 (53) | 12 (18.2) | 19 (28.8) |
|  | Gatifloxacin | 33 (50) | 6 (9.1) | 27 (40.9) |
| Aminoglycosides | Streptomycin | 51 (77.3) | 3 (4.5) | 12 (18.2) |
|  | Kanamycin | 49 (74.2) | 5 (7.6) | 12 (18.2) |
|  | Gentamicin | 26 (39.4) | 9 (13.6) | 31 (47) |
|  | Tobramycin | 27 (40.9) | 8 (12.1) | 31 (47) |
|  | Amikacin | 17 (25.8) | 13 (19.7) | 36 (54.5) |
|  | Netilmicin | 30 (45.45) | 6 (9.1) | 30 (45.45) |
| Tetracyclines | Doxycycline | 66 (100) | 0 (0) | 0 (0) |
|  | Minocycline | 66 (100) | 0 (0) | 0 (0) |
| Folate pathway inhibitors | Trimethoprim/Sulfamethoxazole | 58 (87.9) | 0 (0) | 8 (12.1) |
| Phenicols | Chloramphenicol | 48 (72.7) | 10 (15.2) | 8 (12.1) |
| Phosphonic acids | Fosfomycin | 13 (19.7) | 2 (3) | 51 (77.3) |
| Nitrofurans | Nitrofurantoin | 66 (100) | 0 (0) | 0 (0) |
